# Overview of the User Experience for Snorkeling Mask Designs during the COVID-19 Pandemic

**DOI:** 10.3390/healthcare9020204

**Published:** 2021-02-14

**Authors:** Jacopo Profili, Emilie L. Dubois, Dimitrios Karakitsos, Lucas A. Hof

**Affiliations:** 1 Laboratoire d’Ingénierie de Surface, Centre de Recherche sur les Matériaux Avancés, Département de Génie des Mines, de la Métallurgie et des Matériaux, Université Laval, 1045 Avenue de la Médecine, Quebec City, QC G1V 0A6, Canada; jacopo.profili@crchudequebec.ulaval.ca; 2 Centre de Recherche du CHU de Québec-Université Laval, Hôpital St-François d’Assise, 10 rue de l’Espinay, Quebec City, QC G1L 3L5, Canada; 3 Agence IMPAKT Scientifik Inc., 435 Chemin Sainte-Foy, Quebec City, QC G1S 2J2, Canada; emilie@impaktsci.co; 4 Critical Care Department, King Saud Medical City, Riyadh 12746, Saudi Arabia; karakitsosdimitrios@gmail.com; 5 Department of Medicine, University of South Carolina, School of Medicine, Columbia, SC 29209, USA; 6 Critical Care Department, Keck School of Medicine, University of Southern California, Los Angeles, CA 90033, USA; 7 Department of Mechanical Engineering, École de Technologie Supérieure, 1100 rue Notre-Dame Ouest, Montreal, QC H3C 1K3, Canada

**Keywords:** personal protective equipment, non-invasive ventilation systems, rapid prototyping, full-face snorkeling masks, healthcare products, COVID-19 pandemic

## Abstract

During the first wave of the COVID-19 pandemic, industries and academic institutes have collaborated to resolve the worldwide medical supply shortage issues. Innovative designs of 3D-printed items were proposed and developed by the maker community as a temporary solution to address the lack of personal protective equipment. An overview of global ongoing and past initiatives during the COVID-19 pandemic along with their challenges on retrofitting full-face snorkeling masks for healthcare applications such as splash-proof face shields, respirator masks and non-invasive ventilation systems are reported in this contribution. This study reviews these global initiatives and challenges. From our analysis, the present situation highlights the need to build solid networks between healthcare institutes and the different rapid prototyping initiatives. A clear feedback system needs to be implemented to facilitate effective collaboration between engineering (maker) and healthcare teams, to optimize the available human resources, and to achieve adequate product developments responding to the needs of healthcare workers.

## 1. Introduction

Viral diseases have been the cause of serious regional infectious outbreaks and pandemics in the last fifty years (Table 1). The current outbreak of the novel severe acute respiratory syndrome coronavirus 2 (SARS-CoV-2) disease 2019 (COVID-19) pandemic emerged in Wuhan, China in 2019 and resulted in 54 million infections and 1.3 million deaths worldwide thus far [1,2,3]. The highly transmissible SARS-CoV-2 is still spreading across various geographical areas and exhibiting diverse epidemiologic patterns.

Overall, the socioeconomic burden of the imposed travel and trade restrictions due to the current pandemic cannot be underestimated. It was projected that by the end of 2020, the impact solely due to aviation losses might negatively reduce the World Gross Domestic Product up to 1.6%, while job losses may rise to the value of 25–30 million [4].

To slow down the SARS-CoV-2 transmission rate, governments and the World Health Organization (WHO) employed several measures such as personal hand cleaning, application of face masks and social distancing [9]. The extreme surge in demand for specific medical supplies, such as personal protective equipment (PPE) and hospital respiratory equipment (e.g., non-invasive ventilation (NIV) systems), resulted in a drastic shortage worldwide in the early phase of the pandemic [10]. In particular, PPE for healthcare workers (HCWs) such as protective face and respirator masks are still among the most demanded products [11]. The breakdown of the medical supply chain was mainly due to the following factors:

- Decay of stock supplies [12,13,14];
- Monopoly of the medical supply production (i.e., before the pandemic, half of the face masks were produced in China, where production was reduced drastically due to lockdowns and supply chain disruptions) [15,16];
- Limitations of commercial exchanges caused by transport regulations [17,18];
- Increase of product prices due to greater demand [17,19];
- Lack of flexibility and agility of product supply chains in times of crisis [20,21,22].

In this context, different initiatives from industry, academic institutes and the community strongly contributed to resolve this medical supply shortage issue [23]. Several authorities such as the United States Food and Drug Administration (FDA), the United Kingdom National Health Service (NHS) and Health Canada adopted a flexible policy to address issues related to the global medical supply chain [24,25,26]. Hence, the standard of care shifted towards the priority to preserve life during the COVID-19 crisis. This resulted in strong horizontal innovation, regardless of the need for standardized medical products, calling for rapid design and production. New collaborative manufacturing models [27], focused on local rapid fabrication of most urgently needed medical supplies (e.g., patient ventilation systems and PPE such as face or respiratory masks), were launched, integrating additive manufacturing (AM) (also referred to as three-dimensional (3D) printing) to create both components of medical devices and full-end products [28]. Many international manufacturing challenges (i.e., competitions) were also proposed by several organizations and health expert assemblies (Table 2).

The current pandemic situation revealed the need to build solid networks between “makers” (academic laboratories, industry, individuals, maker communities) and “users” (hospitals, healthcare workers, individuals) for effective production of relevant medical supplies meeting the demand for HCWs [34]. Among the different projects during the pandemic, a significant part focused on the modification of full-face snorkeling masks to develop personal protective equipment (PPE) [35]. Three-dimensional (3D)-printed products were either adapted ‘from’ existing commercial products or ‘to’ existing commercial products due to the difficulty of obtaining medical certification and urgent demand.

**Figure 1:**
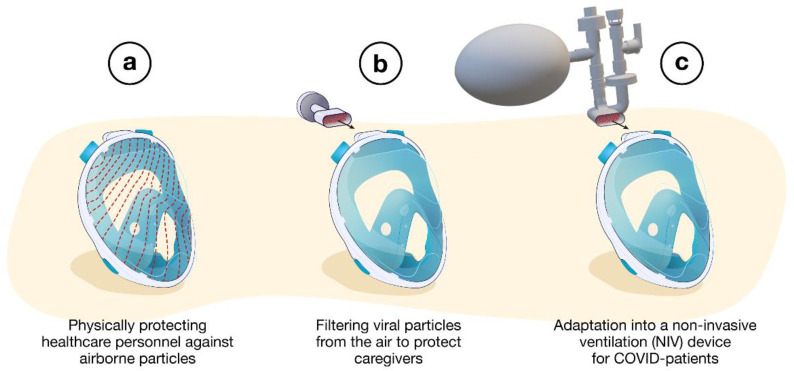
Different strategies used to redesign snorkeling masks for medical use during the COVID-19 pandemic: (**a**) splash-proof face shields, (**b**) respirator masks, (**c**) non-invasive ventilation devices.

The aim of this investigation is to review the global initiatives on the use of snorkeling masks during the COVID-19 pandemic. Full-face snorkeling masks have been used to ensure physical or biological protection against the spread of the virus [36]. In addition, the use of full-face snorkeling masks as non-invasive ventilation (NIV) devices for patient oxygenation (ventilation) has been reported [37,38,39]. In this case, a 3D-printed valve connects the mask to a standard non-invasive ventilation system working with either a bilevel or continuous positive airway pressure (BPAP or CPAP mode, respectively) [40,41]. Such a retrofitted device has been proposed to help non-critical patients, leaving the use of invasive ventilators for the most critical patient cases [40]. Figure 1 summarizes the different user strategies proposed for snorkeling mask use in health care. These deployed strategies include: (1) physically protecting HCWs against airborne particles (face shield); (2) filtering viral particles from air to protect HCWs (respirator mask); and (3) adaptation towards a NIV system for COVID-19 patients (NIV devices) Figure 1a–c.

Although many initiatives were reported online, there is a lack of comprehensive data related to design and fabrication details as well as geographical location of the developed initiatives on retrofitting full-face snorkeling masks. In this communication, we aim to address this issue by providing an overview of ongoing and past initiatives along with their challenges on retrofitting full-face snorkeling masks for healthcare applications during the COVID-19 pandemic. This contribution concentrates on reviewing developed “connectors” (also referred to as “valves” by different makers) to repurpose full-face snorkeling masks for healthcare applications, such as splash-proof face shields, respirator masks and NIV systems (Figure 1). This study provides information as well on fabrication and use of 3D-printed connectors to repurpose snorkeling masks. This information has been mostly retrieved from healthcare professionals and engineers reporting in scientific articles. Additional information has been collected from the different initiatives published online by the different manufacturers and maker communities. Finally, a preliminary set of recommendations and practice guidelines is proposed.

## 2. Material and Methods

An initial search in online databases (Web of Knowledge, Science Direct, Scopus and Google Scholar) was conducted between March and October 2020, to identify academic literature detailing the use and modifications of snorkeling masks during the COVID-19 pandemic. Additional searches followed until early December 2020. The terms “snorkeling or snorkel mask and diving mask” were used in combination with the terms “COVID” and “3Dprinting” during the searches in these databases. Additional criteria (e.g., initiative’s names) were also set on a daily basis to compensate the limited sources available. However, it must be noted that a large part of the developments and initiatives could be found only on websites, as most initiatives focused on rapid dissemination of their findings and results. For this reason, this review combined scientific articles as well as online data retrieved from reliable public agencies or institutions. All articles and information were rigorously verified on relevance and quality before downloading and use as an information source. Considering the rapid evolution of the COVID-19 pandemic, it is important to note that many documents are rapidly published, and information was updated on a daily basis. The present work reports the innovative designs of 3D-printed items, which were adapted to existing non-medical masks [42]. Instead of fabricating full respirator masks from scratch, retrofitting common full-face snorkeling masks was explored by the community [43,44]. Several innovative industrial, academic and not-for-profit organization global initiatives on snorkeling mask repurposing were proposed during the first wave of the pandemic. These initiatives [45,46,47,48,49,50,51,52,53,54,55,56,57,58,59,60,61,62,63,64,65,66,67,68,69,70,71,72] are detailed in Section 4 *Discussion* and presented on a worldmap grouped by their geographical location. It should be noted that these initiatives are mainly located in North America and Europe as they host the largest snorkeling mask companies.

## 3. Different User Strategies for Retrofitted Snorkeling Masks

The proposed user strategies for using snorkeling masks in health care are summarized in Figure 1, including their use as a splash-proof face shield, respirator mask and NIV device. These user strategies are presented and discussed in more detail in the following Section 3.1, Section 3.2, and Section 3.3.

### 3.1. Splash-Proof Face Shields

In the first case (Figure 1a), the mask is used as a splash-proof face shield and no additional printed item is necessary. The main function of the mask is to limit the flow of relatively large airborne particles (e.g., large droplets of infected saliva (submillimeter sizes)) [73] directed to the face. Of note, splash-proof face shields are less protective of infectious aerosols (<5 µm) [74], as these can be inhaled either by the space between the shield and face or by the inhalation tubes in the case of using a snorkeling mask as splash-proof face shield. However, the advantage of using snorkeling masks is full-face coverage (nose, mouth and eyes), compared to standard face shields, thus limiting self-contamination by hand-to-eye contact. The firm fixation of the snorkeling masks to the head ensures protection during rapid movements (e.g., falling, running, etc.). However, a few practical aspects could limit their use. Snorkeling masks remain uncomfortable (e.g., relatively high resistance for breathing, condensation within the mask) compared to classic face shields. Proper mask handling [35] (e.g., putting on and taking off the mask must be followed by a cleaning procedure) is another concern to comply with the WHO recommendations [75]. Lawrence et al. [36] evaluated the extent of droplet contamination during mastoid drilling in a preclinical model. The authors reported virus contamination extended for over 2 m from the drill site. While, in this work, the protection, the comfort and the breathability with the modified snorkeling mask were validated as adequate/good, some problems were observed. For example, a standard full-face respirator appeared to be more effective for HCW–patient communication compared to a modified full-face snorkel mask [36]. Therefore, most of the participating surgeons stated that they would not personally use the modified snorkeling mask in future treatments/work. Communication issues between coworkers while wearing snorkeling masks were also reported by Palmeiro et al. [28]. To overcome this problem, solutions were proposed, including C-Voice Masks, manufactured by an Italian company named Siropack and the Industrial Engineering Department of the University of Bologna [76]. This device was designed with an audio amplification system to facilitate the communication between HCWs. Similarly, Krool et al. created a mobile application to transmit the voice from a wireless microphone inside the mask to speakers in the environment of use [77].

### 3.2. Respirator Masks

**Figure 2:**
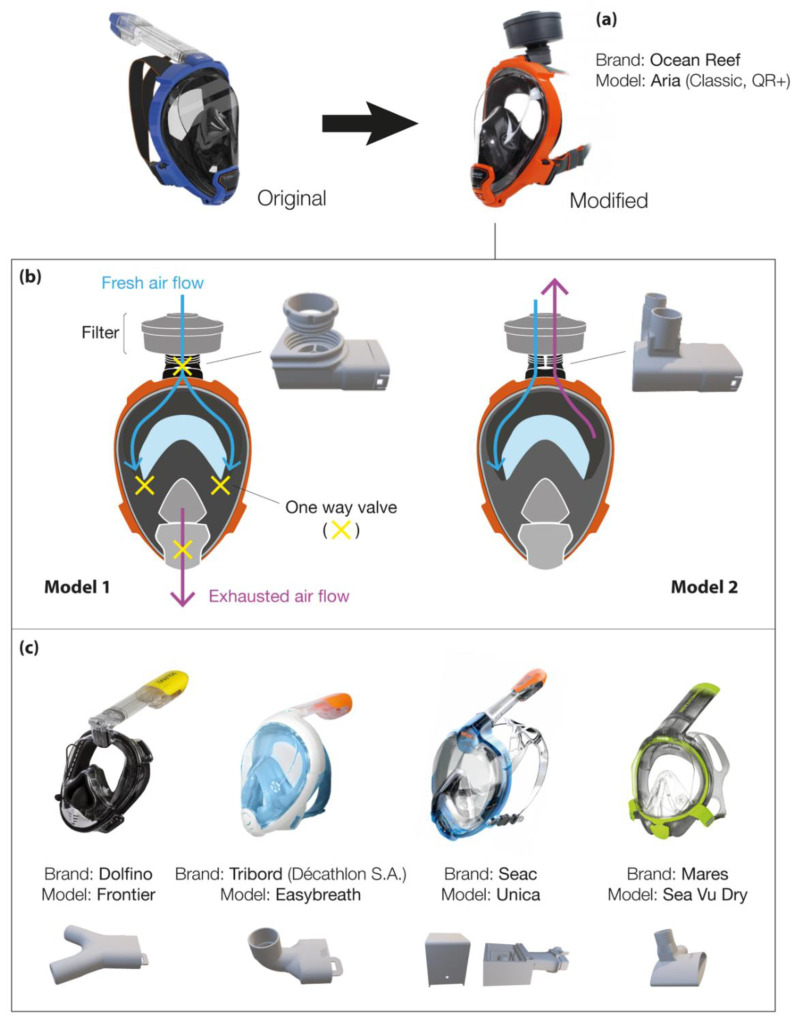
(**a**) Modifications of Ocean Reef’s masks using 3D-printed components; (**b**) different respirator modes obtained with the Ocean Reef’s masks; (**c**) most common masks studied by the community during the COVID-19 pandemic.

In the second case (Figure 1b), the snorkeling mask is used as a full-face particulate-filtering face-piece respirator. The 3D-printed adapters replace the snorkel exhale channel usually located at the top of the mask. These respirators are known as N95 masks when they conform to Occupational Safety and Health (OSH) standards. Quality N95 filter material is scarce as it should be composed of non-woven melt blown fabric, which is not readily available in many countries. As a result, makeshift masks are equipped with all sorts of alternative filters (i.e., cheap surgical masks, Halyard H600 drapes, generic filters with some high-efficiency particulate air (HEPA) classification). Figure 2a highlights the modifications of the Ocean Reef snorkeling mask, an open source project developed by the Italian company Mestel Safety (Ocean Reef Group) [78]. The company offered the possibility to buy the patented connectors at low prices to help owners convert their snorkeling masks. If needed, 3D printing design files were shared online for non-commercial use [79]. Two coupling adapter prototypes were proposed, which were suitable for different conditions of application (Figure 2b).

The first adapter was for general use, where a sterile environment was not required. In this case, the one-way silicone valve (i.e., mushroom valves), present near the mouth/chin area, allows exhausted air to be expelled from the lower part of the mask. This design corresponds more to a respirator mask with a breather valve, which protects the user but does not filter its exhalations [80]. The second adapter filters both the inhaled and the exhaled air and is specifically designed for HCWs. The 3D-printed connector has two separated circuits: one for the intake and one for the exhaust. The one-way silicone valve near the mouth/chin area is blocked (usually with tape or glued shut). An additional breathing valve is placed in the exhaust connector to limit the return air from filters and the dead air space in the mask. Nicholson et al. studied the influence of three experimental configurations of the snorkel mask (i.e., removing the mushroom valve, blocking the valve exhaust, normal conditions) during a medical standard fit test [81]. All analyses were performed following the United States Occupational Safety and Health Administration (OSHA) 29CFR1910 standard [82]. Although the qualitative analysis exceeded the OSHA requirements for all conditions, significant differences were observed between blocking and opening the valve exhaust. This suggested a reduced protection by modified masks when the mushroom valve is in place. In addition, Krool et al. demonstrated that the custom adapter and the chin valve did not generate significant leaks [77]. The authors showed that fit factors corresponded to the requirement for elastomeric half-masks. Another study also confirmed that the modified mask passed the OSHA N95 respirator fit test requirements (fit factors should exceed 100) as a fit factor of 142 was obtained and neither hypercapnia nor hypoxemia occurred. [83] Additional information can also be found from the work of Tack et al. [84]. The authors tested the modified snorkeling mask on 40 healthcare workers (COVID-19-free) during 2 h of normal work. The retrieved data did not show significant changes for blood gas analysis after 120 min. Masks were well within the required tolerances, although after 2 h, 17% of the participants reported some difficulties (ratings of perceived exertion (RPE) scored at 5/10). It is important to note that over the last months, several adaptors were developed due to the wide variety of full-face snorkeling mask brands [77]. This has increased the number of immediate transformable protection equipment to two million pieces (considering the available stock to date) [78]. The quality and the price of these full-face masks vary depending on the geographical area and the brand. For example, in the USA, prices range from 20 to 90 USD for a single mask, with a mean cost of approximately 56 USD [77]. Figure 2c outlines the four most common brands studied by the community during the COVID-19 pandemic, together with their developed connectors to use these snorkeling masks as respirator masks. Vicini et al. tested five different modified masks during otolaryngological surgery and in anesthesia procedures on COVID-19-free healthcare professionals [85]. The deployed modified devices were adapted from the following brands:

- Subea Easybreath (Decathlon, France);
- Seac Unica (Seac Sub, Italy);
- Siropack C-Voice, (Siropack, Italy);
- Ocean Reef Aria QR+ (Mestel Safety, Italy).

The authors concluded that a more customized mask is necessary to fulfill the medical and surgical needs. As an example, the authors suggest modifying the system to allow the use of personal glasses inside the mask, including a microphone/loudspeaker system and integrating a lamp as well as a magnifying loupe system, all necessary for well-functioning of most medical procedures. Similarly, Dalla et al. reported a study on modified Ocean Reef snorkel masks [86]. Over 22 masks were fit-tested on healthy individuals. The authors report that 86.9% (n = 459) of the models that passed qualitative fit tests (QLFT) also passed quantitative fit testing. Among the wide variety of proposed designs for snorkeling mask retrofitting for use as PPE, pioneering work was conducted by the Prakash team at Stanford, together with Dolfino Frontier^®^ designed mask connectors integrating standard commercial filters (medical or industrial grade) [77]. To use industrial National Institute for Occupational Safety and Health (NIOSH)-approved filters, an additional modular component was designed, and a proper gasket was mounted in the assembly. The results obtained from fit tests changed depending on the analyzed area in the mask. [77]. The same results were obtained when the sampling tube was placed close to the mouth or in the eyes chamber. In contrast, significant higher leaks were found when the tube was connected to the 3D-printed adapter. The authors concluded that leaks depend on how well the mask fits the wearer’s face. Thus, results vary depending on the combination of face and mask shapes. Regarding materials, Prakash et al. report working mostly with a biocompatible polyethylene resin (Carbon RPU-70 from PrinterPrezz [87]) [77]. The Grey Pro Resin (from Formlabs) was also used due to its good mechanical performance [88]. However, the former had issues regarding achieved tolerances, so a careful check during the assembling steps was suggested. Moreover, fused deposition modeling (FDM) may result in porous connectors, which remain dangerous in the presence of viral gases (video [89]). Previous studies identified key parameters affecting the final quality of different 3D-printed objects [90], such as the wall permeability depending on the geometric shape, the filament feed rate and the predefined (G-code) wall structure. Although most of the investigations suggested that performance and efficiency of the tested masks were comparable or exceeded the N95 standard, all authors recommended caution as the adaptors were not certified for medical use and companies as well as inventors did not guarantee the safety of the protection device (mask + connector). Last April, the FDA issued an updated guidance addressing these issues [91]: when alternatives, such as FDA-cleared masks or respirators, are unavailable, individuals (including healthcare professionals) might improvise PPE. Similarly, the National Medicines Safety Agency, the French version of the FDA (ANSM), authorized the distribution of adapters in France until 31 May 2020, as the material ensuring the same protection guarantees and meeting the compliance requirements was not available [92]. Another preliminary study conducted in Canada outlined the challenges for retrofitting N95 filters on snorkeling masks [93]. Based on the Centers for Disease Control and Prevention (CDC) Guidelines on Crisis/Alternate Strategies for PPE decisions in the setting of N95 respirator shortages [94], the modified snorkeling masks can be considered as “healthcare personal use of non-NIOSH-approved masks or homemade masks”.

### 3.3. Non-Invasive Ventilation Devices

**Figure 3:**
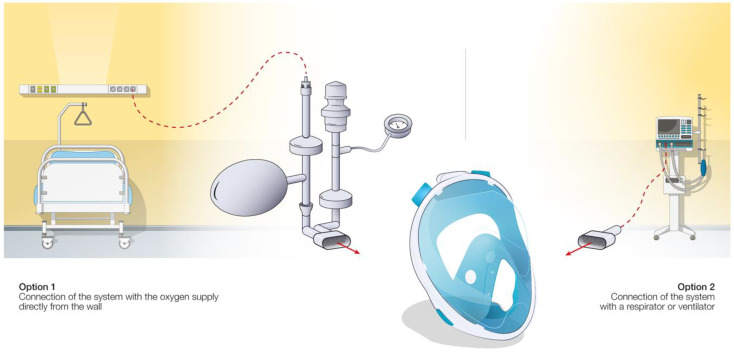
Different configurations of snorkeling mask usage and developed connectors as non-invasive ventilation devices.

In the third case (Figure 1c), standard snorkeling masks have been modified into non-invasive ventilation (NIV) devices for the treatment of acute hypoxemic respiratory failure of COVID-19 patients [95]. These modified masks (MM), by adequately replacing the unexpected shortage of the standard medical ventilation devices and masks, ensured a positive airway pressure during the entire respiration cycle and reduced the intubation rates [96]. The MM requires disposable tubing components along with 3D-printed connectors. Different configurations have been proposed as presented in Figure 3.

**Figure 4:**
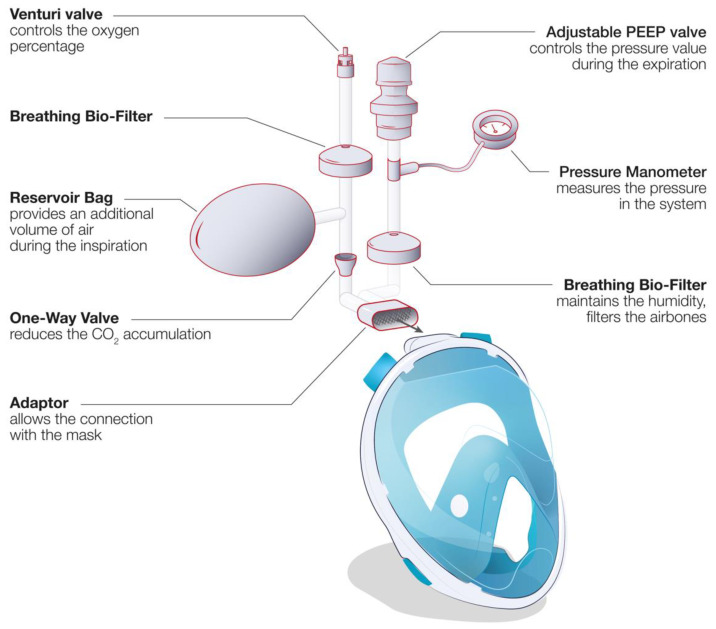
Full-face snorkeling mask adapted for use as a non-invasive ventilation device: schematic of the different components when using a Venturi valve and function of each element.

In a conventional ventilation method, the standard medical mask is connected directly to a CPAP module (e.g., WhisperFlow 2–60 from Respironics). This situation of direct connection of a mask to a respirator/ventilator device is outlined in Figure 3, option 2, for the case of retrofitted snorkeling modified masks (MM) using the developed (mostly 3D-printed) connectors. Alternatively, the system can be connected directly to the pressure-regulated oxygen source of the hospital (deployed pressures are usually between 3.5 and 50 bars before the pressure regulator) and an oxygen air mixer can be placed between the mask tubing and the source of oxygen (e.g., EasyMIX^®^ from Flow-Meter S.p.A). Some authors also reported the possibility to connect the system directly to the oxygen source without any flowmeter [97], having the oxygen supply controlled by a Venturi valve (e.g., 0060000 from Intersurgical) typically used during oxygenation therapy. Hence, the tubing and the design of the NIV MM were adapted to ensure correct operation of the system (Figure 3, option 1). The majority of hospitalized COVID-19 patients require high fraction of inspired oxygen (FiO_2_) during NIV [98]. However, the highest FiO_2_ that can be obtained via commercial Venturi valves is usually limited to 60%. In order to increase the FiO_2_, a modified device using a Venturi valve with a secondary inlet was suggested [99], as shown in Figure 4.

Most of the MM designs used commercial medical tubes (e.g., 1528000, 22 mm Flextube™, 1.6 m long, Intersurgical) to connect the mask to an available gas supply. An adjustable positive end-expiratory pressure (PEEP) valve (e.g., item n. 2226000, 2.5–20 cm H_2_O, 22 m, Intersurgical) was also integrated to control the positive pressure inside the mask. To limit the spread of the virus outside the closed circuit, two breathing filters (e.g., item n. 1544007 Clear Guard™3, Intersurgical) were positioned at the entrance and exit of the circuit. Some of the MMs used a reservoir bag placed in the inlet (e.g., 2820000 Reservoir bag, 2 L with antiocclusion cage mount, 22 F neck, Intersurgical) to increase the air volume and pressure during the first seconds of inspiration. A one-way directional valve (e.g., item n. 1921000 22M flow in, 22F flow out, Intersurgical) was often placed between the mask and the entrance of the gas flow to reduce the dead space. This one-way directional valve is placed after the bag reservoir to decrease any CO_2_ accumulation during the respiratory cycle. A disposable manometer (e.g., item n. 7162000, Intersurgical) could also be connected to the circuit to measure the internal pressure for improved system control. Different commercial adaptors were adopted (i.e., 1568000 straight connector 22F, 6 mm oxygen stem, Intersurgical) to fit the different items together. Actual case studies conducted on virus-infected patients are scarce in literature. Wagner et al. [38] reported the use of a modified snorkeling mask as a NIV device during spontaneous breathing on a female patient, 56 years old, with SARS-CoV-2 infection resulting in the coronavirus disease (COVID-19), and having systemic arterial hypertension and obesity. The mask was used two times/day for 40–60 min, combined to a mechanical ventilator until day 12 of hospitalization. This work demonstrated that the snorkeling mask during spontaneous breathing is effective to prevent orotracheal intubation (i.e., the use of invasive ventilation system). However, a comparative study with a commercial medical mask and a larger number of cases are necessary to support and statistically validate this study. Another study was performed by Bibiano-Guillen et al. at a Spanish medical center, Hospital Universitario Infanta Leonor of Madrid [39]. They deployed ventilation therapy using modified snorkeling masks, which were tested interruptedly on 25 patients having acute respiratory syndrome secondary to SARS-CoV-2 infection. In all studies, no hypercapnia or respiratory acidosis was observed in any patient, meaning that rebreathing did not take place. For this reason, the authors conclude that the use of modified snorkeling masks “complies with clinical requirements to be produced in other centers with limited resources, especially in developing countries” if there is a “deep shortage of certified alternatives” but the users must be “aware of the limitations”. To date, additional tests, made with a more statistical and medical methodology, seem necessary to highlight the relevance and the safety of this approach.

## 4. Discussion

**Figure 5:**
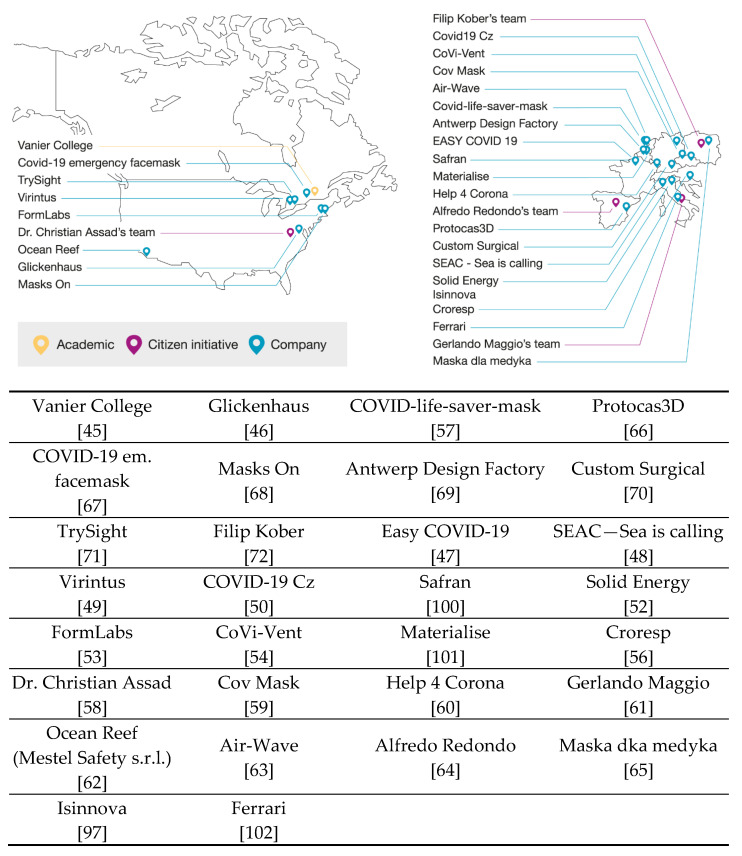
World map representing different initiatives in North America and Europe on manufacturing ecosystems for modified mask (MM) fabrication to use in healthcare institutes during the supply shortage in the first wave of the COVID-19 pandemic [45,46,47,48,49,50,52,53,54,56,57,58,59,60,61,62,63,64,65,66,67,68,69,70,71,72,97,100,101,102].

The use of 3D printing for manufacturing medical devices is a flexible and rapid approach to overcome the supply shortages in various healthcare and community settings. The main advantage consists in the availability of ready-to-use products that are in stock (here: full-face snorkeling masks). The geographical origin (including references) of the main snorkeling mask healthcare retrofitting initiatives over the past months have been highlighted in Figure 5. At this stage, it is important to note that most of the initiatives are in in North America and Europe. This is most probably related to the geographical location of the most important snorkel mask manufacturers. The different initiatives are categorized as “Academic” (identified in yellow), “Citizen initiatives” (identified in violet) and “Company” (identified in blue) activities, providing a complete overview, to the best of our knowledge, of ongoing and past work on use of snorkeling masks to assist HCWs during the pandemic.

Despite the geographical distribution of these collaborative works, it remains important to note that no cost/benefit analysis has been done yet. The capacity to revert the production of standard engineering-related commercial products into medical devices (e.g., connectors for retrofitting) is a time-consuming process which can potentially lead to geometrical/dimensional errors, resulting in lower product performance (e.g., reduced mechanical properties, safety issues). Hence, the following aspects should be considered when producing high quality and safe 3D-printed products:

- Stock availability/price of commercial masks,
- Availability and price of the commercial product computer-aided design (CAD) files,
- Designs covered by copyright or patent,
- Access to dimensional measurement tools.

It is also important to note that among the different 3D printing technologies, Fused Deposition Modeling (FDM) has been largely used during the pandemic [103]. This 3D printing technology remains relatively low cost and its printing speed is relatively high. However, one must consider that FDM 3D printing processes are in most cases (1) not adapted for mass production of objects, as it is a serial process, and (2) used for rapid prototyping with low finishing quality. Therefore, other polymer printing technologies (e.g., resin-based) such as selective layer adhesion (SLA), digital light processing (DLP) and PolyJet or powder-based technologies, such as selective layer adhesion (SLS) and binder jetting, can be also used [104]. These techniques provide an improvement of the 3D printing quality by elimination of microscopic structural defects. However, these approaches are much more expensive in equipment investment and material use and require higher level technical skills compared to FDM technology and therefore are less cost-effective and less easily deployable by maker communities, service providers or HCWs for connector (valve) production. Once mass production of proven designs is required, more conventional polymer manufacturing processes such as injection molding are preferred to provide best results, considering cost and final quality of the product (i.e., low porosity to reduce leakage and contamination, high reproducibility and high dimensional tolerances) [105,106]. In this context, it is important to note that AM can have some major limitations compared to conventional fabrication methods, which are generally well-adapted for mass production of medical devices [107]. Therefore, it is recommended to use AM to rapidly support industrial supply chains during shortages on conventional production instead of replacing them.

When the first COVID-19 pandemic wave took place, many manufacturers declared on their websites that those devices were not subjected to a medical peer review and therefore not yet medically certified. Currently, minimal data support the use of uncertified medical devices in healthcare institutions. To our knowledge, an unofficial list (i.e., assessment by 25 doctors) was reported by Isinnova et al. [108]. Up to now, few clinical studies have evaluated the products obtained by AM or the NM’s performance for NIV or PPE safety for HCWs. Considering the airway management guidelines, different documents and studies have been published from public institutes since the beginning of the pandemic [109], which are mostly updated on a regular basis to summarize a clear and up-to-date guidance to follow. In general, it is important to provide equipment, approved by the responsible government organization, to the patient and HCWs, to protect their health. In case of shortage, the adapted snorkeling masks with 3D-printed connectors must have an efficient filtering system, meeting at least standard Filtering Face Piece class 3 (FFP3) masks. This means that products must be tested before their mass production to minimally follow the guidelines provided by WHO [110]. Three main safety concerns must be considered here:

(1) Is the CO_2_ retention low to ensure safety after the adaptations?
(2) Is the protection sufficient against the viral load of SARS-CoV-2?
(3) Is the pressure during the expiration and the expiration safe in NIV devices?

In this context, Tack et al. concluded that CO_2_ retention inside the mask has no significant impact on arterial blood gas parameters during the test [84]. Nevertheless, additionally, tests are necessary to monitor the effects after 2 h and with unhealthy (i.e., virus-infected) individuals. Similarly, Vicini et al. monitored the partial pressure of oxygen (PO_2_) and carbon dioxide (PCO_2_) values dissolved in the blood on a modified Ocean Reef’s mask during surgical procedures [85]. The PCO_2_ were within normal ranges and the parameters indicate correct breathing and normal air circulation in the mask.

However, contradictory studies are published concerning the suitability to use these types of MMs in the medical field. To date, this results in a lack of confidence in using these MMs from HCWs [111]. For example, Lawrence et al. observed a significant rise in the fractional concentration of inspired CO_2_ (FiCO_2_) for the full-face snorkel mask values. Their CO_2_ levels were higher than 1% after 30 min, which is above the health and safety regulations for respiratory PPE [36]. In addition, Greig et al. concluded that snorkel masks are poorly suited as a PPE [112]. Even if the authors found that no changes in gas composition that were recorded over 20 min (i.e., fractional concentration of inspired O_2_ FIO_2_, end-tidal oxygen (ETO_2_) and end-tidal CO_2_ (ETCO_2_) showed no changes), the modified mask failed other quantitative tests (this work has been limited to only a single user and a single combination of adaptor and mask). Germonpre et al. reported that a small initial increase of CO_2_ is observed at the end of an exhaled breath (i.e., ETCO_2_). However, this increase remains within the physiological limits and the authors conclude that 3D-printed adaptors used with commercial snorkeling masks (Subea, Aqualung, Seac, Cressi and Ocean Reef) are safer, have more flexibility and reliability than makeshift adaptations [113].

It is also important to note that most deployed filters have a bacterial and viral filtration efficiency of 99.999%. While different configurations of filtration exist (i.e., electrostatic, pleated mechanical), all filters for ventilatory use are equally effective [113]. A review and extensive testing of commercially available filters has been published by Wilkes et al. [114]. Considering the quality of the AM process as well as the (low) accuracy of the printed connectors, it is important to evaluate the sealing of the device, or more specifically, the mask fit. This is measured by either qualitative fit tests (QLFT) or quantitative fit tests (QNFT). The QLFT consists to see if a bitter or sweet scent sprayed outside the mask can be detected by the user. On the other hand, QNFTs measure ratios of aerosols inside and outside the mask. Here, the protection level is often measured by using a particle counter (e.g., TSI PortaCount Pro+ 8038; TSI Incorporated, Shoreview, MN, USA). Germonpre et al. highlight that most snorkeling masks can provide protection levels within acceptable limits comparable to N95/FFP2 or even N99/FFP3 levels [113]. However, dissimilarities have been observed, depending on the used brand and the type of the deployed filter. Schmitt et al. studied the filtering efficiency on a modified Easybreath (Decathlon) mask [115]. In their study, the chin valve was sealed. Filtration efficiencies were measured only for particle sizes lower than 300 nm to cover the size of the virus and small droplets. Their results showed than the modified snorkeling mask can reach the requirements for the FFP2 level. In addition, the devices have good resistance to several cycles of decontamination (autoclaving and ethanol immersion). Mortimer Gierthmuehlen et al. reported a filter efficacy of 85.07% for the modified scuba diving mask by using a scintigraphic camera and a 99mTc-labeled NaCl aerosol [116]. Consider that FFP2 and FFP3 masks must have a mean leakage of maximum 8% and 2% as well as a protective of 95% and 98%, respectively, against a standard formula.

It should also be noted that only a few studies have been reported in the literature concerning the performance of the MM for alveolar recruitment as well as gas exchange when used as a CPAP device. In this context, Landry et al. compared the performances of a snorkel mask and oronasal CPAP on two healthy male volunteers [41]. The experiments were conducted with controlled O_2_ and a positive pressure delivery system. During the experiments, the snorkel mask delivered CPAP levels up to 14 cmH_2_O. However, while CPAP masks have a small decrease in FIO_2_ (0.8%/cmH_2_O) with the increased pressure, the snorkel mask demonstrated stronger decline in FIO_2_. This particularly occurred when CPAP >12 cmH_2_O and this situation corresponded with an increase in leaks. This is particularly problematic in the context of a pandemic as aerosolization of the virus will then occur. In this work, the snorkel mask also demonstrated a higher CO_2_ accumulation (25 mmHg, ~3%) compared to the CPAP mask (5 mmHg, ~0.6%). Similarly, Alberto Noto analyzed the efficiency of an EasyBreath^®^ mask as an emergency respiratory interface for a CPAP system [40]. The authors studied the stability of pressure generated inside the mask with and without a turbine-driven ventilator. The amount of carbon dioxide (CO_2_) rebreathing was also recorded during spontaneously and resistively loaded breathing. Their experiments were made on 10 healthy volunteers with a high-flow system generator. The authors report substantial CO_2_ rebreathing for flow rates below 80 L min^−1^. The highest inspired CO_2_ partial pressure (PiCO_2_) was recorded at 40 L min^−1^ (min flow rate) with CPAP 15 cmH_2_0 (max CPAP level). This was mainly related to the wide dead space (~900 mL) inside the mask. The use of a turbine-driven ventilator or high flows are necessary to wash-out the exhaled CO_2_ completely. Similarly, end-inspiratory to end-expiratory pressure swing, which is related to respiratory effort, was clinically relevant (>3 cmH_2_0) only with a flow rate lower that 80 L min^−1^. The use of a reservoir bag or a threshold PEEP valve showed no improvement. To date, there is no study which reported a complete analysis obtained with unhealthy (i.e., virus-infected) patients. This implies that additional tests, made with a more statistical and medical methodology, seem necessary to highlight the relevance and the safety of this approach in a medical context not impacted by a temporal shortage of commercial products.

A useful approach to guarantee the pertinence of the rapid prototyping (e.g., by 3D printing) of medical supplies is to ensure direct collaboration between designers and HCWs with constant feedback and adequate testing. Arash et al. described a research protocol for rapid iteration of reusable face shield design coupled with clinical feedback and real-world testing [117]. The authors suggested integrating individuals with engineering and manufacturing expertise into governance structures during a pandemic. They also highlighted the importance of established longitudinal relationships between researchers and local fabrication communities. Similarly, Tino et al. recommended maintaining open communication between hospitals and 3D printing communities to ensure the fabrication of safe products [118]. In this regard, the 3D COVID-19 network created in Paris resembles this multidisciplinary approach. Specifically, the University Hospital Trust *Assistance publique – Hôpitaux de Paris* (AP-HP), among the largest hospital systems in the world, created the largest 3D print platform in France (i.e., 60 industrial-grade 3D printers [119]) in collaboration with a 3D printing service provider [120] and a 3D printer reseller [121]. The hospital printed medical equipment, including face shields, masks, electrical syringe pumps, intubation pieces and respirator valves. A dedicated 3D printing platform [122] to support tracking of requests from other local hospitals resulting in an effective production and supply of PPE was also launched. Another effective strategy was reported by Prusa3d [123]: mass production and delivery of over 200,000 face shields in a short period of time was realized; this was made possible thanks to a digital maker platform which facilitated direct communication among various parties (e.g., HCWs) throughout the full project duration. Another asset of the project led by Prusa was the creation of comprehensive maker protocols, i.e., fabrication protocols ensuring highest 3D print quality and guidance on use, and health certification guidelines [124], ensuring proper handling according to WHO recommendations. An industrial “smart factory” was also initiated by Nuovamacut in Italy [125] for the fabrication of 3D medical connectors. This initiative created a temporary collaboration with the local medical community [126] and Mares, a well-known manufacturer of diving equipment [127]. The CAD files (optimized for 3D printing) were shared with another company named Mira Meccanica [128], which made the first functional prototypes in various shapes and versions. Later on, other companies (i.e., Protoflash [129], Pariani [130], FPZ [131], PIUSI [132], Campetella Robotics Center [133], Ferrari [102], Everex [134], SB3 [135] and Innovamec [136]) joined the network to produce connectors at large scale. According to Nuovamacut et al., many Italian hospitals (i.e., Parma, Bologna, Imola, La Spezia, Genova, Rapallo, Brescia, Pinerolo) participated in the project for uniform quality and appropriate clinical use. Another collaboration was developed between École de technologie supérieure (ETS) in Canada and the Centre Hospitalier de l’Université de Montréal Research Centre (CHUM-RC) and the Centre Intégré Universitaire de Santé et de Services Sociaux (CIUSSS) in Montréal. Researchers at ÉTS together with the clinical staff established a local citywide maker community providing 3D printing services for medical and non-medical purposes [137,138,139]. Several websites were launched to collect information on such local initiatives from different sources during the pandemic. As an example, a government-supported Canadian, provincial website listed all local initiatives for the development of PPE during the first wave of the COVID-19 pandemic [140].

## 5. Conclusions

In conclusion, the integration of AM/3D printing for new medical device production is a flexible and attractive solution to counteract the needs of HCWs and patients during a pandemic. Small-scale three-dimensional (3D) design and printing laboratories have been widely used during the pandemic to increase the local production by parallelizing processes and sharing their resources. As highlighted in this work, many global initiatives have been supported by the maker and academic communities. Specifically, retrofitted snorkeling mask for usage as PPE or oxygen respiratory devices has been possible thanks to the development of 3D-printed connectors (also referred to as “valves”). Different 3D designs, prototypes and knowledge of retrofitting full-face snorkeling masks have been shared online during the pandemic. From a technical point of view, different parameters should be considered to ensure the advantage of employing 3D-printed products: (1) The final cost should be reasonable to guarantee the manufacture at large scale; (2) geographical availability of consumable materials and printers should be optimized as hospitals have often centralized supply demand; and (3) the final printed product must demonstrate a medical efficiency comparable to commercial items with similar features. Several concerns on the quality of the manufactured products (connectors) were raised when using the AM method and care should be taken in using the conditions to realize best connector–mask connections. To date, the safety of most proposed innovative designs remains under examination. For this reason, collaboration between researchers and medical industries along with proper standardization of new medical devices are prerequisites to establish “living lab” approaches. A proper and structured approach must be followed by all the involved parties, both on the engineering manufacturing side and the medical teams. A clear feedback system also must be implemented, ideally with a small ‘in situ’ lab in the medical institution, facilitating the collaboration between the involved teams and optimizing the available human resources. This is particularly true for the use of MM as NIV devices. Indeed, as highlighted by Noto et al. [40]: “The modified mask should be used for emergency use to deliver CPAP only under strict constraints, using a high-flow generator at a flow rate greater than min^−1^, 80 L or with a high-performance turbine-driven ventilator”.

The presented study provides a comprehensive review on ongoing and past initiatives on development of urgently needed PPE and other healthcare-related products during the first wave of the COVID-19 pandemic, to facilitate knowledge dissemination aiming to accelerate effective use of rapid prototyping in the context of pandemics.

## Figures and Tables

**Table 1:** List of the most important viral diseases observed in the last fifty years.

Name	Duration	Deaths	Ref.
SARS ^i^	2002–2003	774	[5]
Swine flu ^ii^	2009–2010	280,000	[6]
Ebola ^iii^	1976–present	12,950	[7]
HIV ^iv^	1981–present	>5 million	[8]
MERS ^v^	2012–present	850	[5]

^i^ Severe acute respiratory syndrome, ^ii^ influenza pandemic, ^iii^ Ebola virus disease (EVD), ^iv^ human immunodeficiency viruses, ^v^ Middle East respiratory syndrome.

**Table 2:** List of international manufacturing challenges in the COVID-19 era.

Name of the Challenge	Organization	Ref
COVID-19 Detect & Protect Challenge	United Nations Development Program	[29]
Code Life Ventilator Challenge	Agorize	[30]
Reusable Face Masks Challenge	Ennomotive	[31]
Mechanical Ventilators Challenge	Ennomotive	[32]
Manufacturing Ventilators Challenge	Ennomotive	[33]

## Data Availability

The data presented in this study are available throughout the article.
