# Correction: Profili et al. Overview of the User Experience for Snorkeling Mask Designs during the COVID-19 Pandemic. *Healthcare* 2021, *9*, 204

**DOI:** 10.3390/healthcare9070817

**Published:** 2021-06-28

**Authors:** Jacopo Profili, Emilie L. Dubois, Dimitrios Karakitsos, Lucas A. Hof

**Affiliations:** 1 Centre de Recherche sur les Matériaux Avancés, Laboratoire d’Ingénierie de Surface, Département de génie des mines, de la Métallurgie et des Matériaux, Université Laval, 1045 avenue de la Médecine, Quebec City, QC G1V 0A6, Canada; jacopo.profili@crchudequebec.ulaval.ca; 2 Centre de recherche du CHU de Québec-Université Laval, Hôpital St-François d’Assise, 10 rue de l’Espinay, Quebec City, QC G1L 3L5, Canada; 3 Agence IMPAKT Scientifik Inc., 435 chemin Sainte-Foy, Quebec City, QC G1S 2J2, Canada; emilie@impaktsci.co; 4 Critical Care Department, King Saud Medical City, Riyadh 12746, Saudi Arabia; karakitsosdimitrios@gmail.com; 5 Department of Medicine, School of Medicine, University of South Carolina, Columbia, SC 29209, USA; 6 Critical Care Department, Keck School of Medicine, University of Southern California, Los Angeles, CA 90033, USA; 7 Department of Mechanical Engineering, École de technologie supérieure, 1100 rue Notre-Dame Ouest, Montreal, QC H3C 1K3, Canada

The authors would like to make the following corrections to the published paper [1]. The changes are as follows:

1. Change the reference 45 to “Sébastien, P.; Tatiana, R.; Nikolay, M.; Patricia, M. On the Use of Venturi Valves to Control Oxygen Supplementation During Positive Airway Pressure Support With ScubPAP Circuits. *Zenodo* **2020**, *7*. doi:10.5281/zenodo.4310319”.

2. Replace the sentence in “Section 4. Discussion” in page of 10: “The different initiatives are categorized as “Academic” (identified in yellow), “Citizen initiatives” (identified in violet) and “Company” (identified in blue) activities, providing a complete overview, to the best of our knowledge, of ongoing and past work on use of snorkeling masks to assist HCWs during the pandemic” with “The different initiatives are categorized as “Citizen initiatives” (identified in violet) and “Company” (identified in blue) activities, providing a complete overview, to the best of our knowledge, of ongoing and past work on use of snorkeling masks to assist HCWs during the pandemic”.

3. Figure 5 should be replaced with the following.

The authors would like to apologize for any inconvenience caused. The changes do not affect the scientific results. The manuscript will be updated, and the original will remain online on the article webpage.

## Figures and Tables

**Figure 5:**
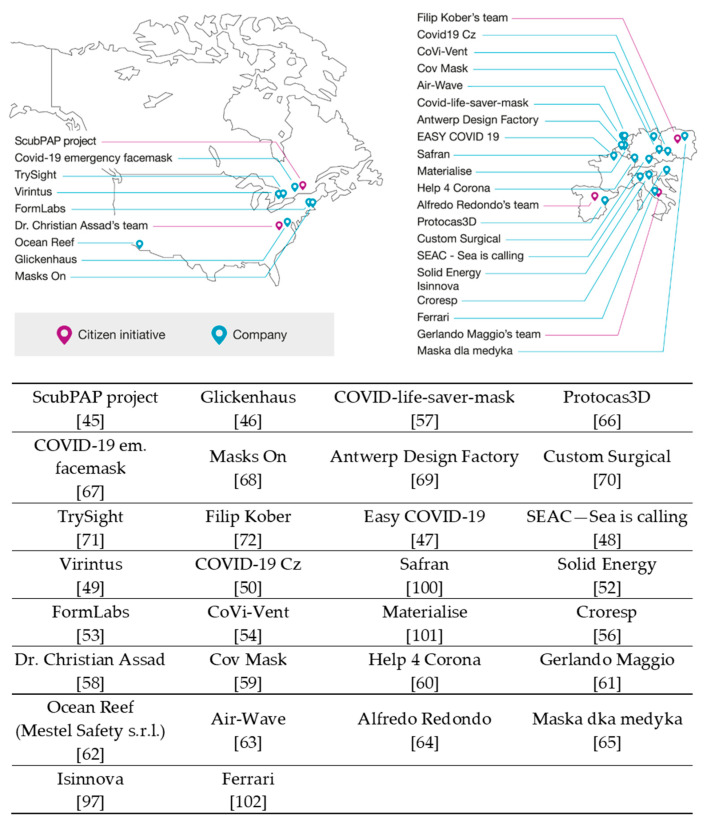
World map representing different initiatives in North America and Europe for manufacturing ecosystems for modified mask (MM) fabrication for use in healthcare institutes during the supply shortage in the first wave of the COVID-19 pandemic [45–50,52–54,56–72,97,100–102].
